# Pilot randomised controlled trial on the feasibility and intervention satisfaction with an educational group programme for adults with attention deficit hyperactivity disorder and their caregivers

**DOI:** 10.1186/s12888-025-06570-4

**Published:** 2025-02-20

**Authors:** Tatiana Skliarova, Rolf W. Gråwe, Jonas Vaag, Arthur Mandahl, Inger Kolltveit, Ina Løvås, Terje Torgersen, Mariela L. Lara-Cabrera

**Affiliations:** 1 Department of Mental Health, Faculty of Medicine and Health Sciences, Norwegian University of Science and Technology (NTNU), Trondheim, Norway; 2 Department of Mental Health Care, St. Olavs University Hospital, Trondheim, Norway; 3 Department of Psychology, Inland University of Applied Sciences, Lillehammer, Norway; 4 Vårres Regional User-Led Centre Mid-Norway, Trondheim, Norway; 5 Department of Mental Healthcare, St. Olavs Hospital, Nidelv Community Mental Health Centre, Trondheim University Hospital, Trondheim, Norway

**Keywords:** Acceptability, Adult attention deficit hyperactivity disorder (ADHD), Caregivers, Patient satisfaction, Patient education, Peer-cofacilitated education, Person-centred care, Psychoeducation, Quality of life (QoL), Randomised controlled trial (RCT), Self-efficacy

## Abstract

**Background:**

This pilot randomised controlled trial (RCT) study presents a patient-centred educational group programme collaboratively developed with user representatives and caregivers from two organisations and health personnel from a community mental health centre (CMHC). The objectives were to investigate the feasibility of the psychoeducational programme and the satisfaction of outpatients and caregivers with programme participation. This RCT explored the potential benefits of this innovative psychoeducational programme for adults recently diagnosed with attention deficit hyperactivity disorder (ADHD).

**Methods:**

This pilot study employed a two-arm RCT design and recruited 60 outpatients from a Norwegian CMHC. The CMHC clinicians offered the intervention group (IG) participants a two-session, patient-centred, peer-cofacilitated programme along with the standard treatment. The control group (CG) received the standard treatment and a 1-h individual informational session during the same period. The recruitment, attendance, and dropout rate indicators were assessed for feasibility. Acceptability was assessed via the Client Satisfaction Questionnaire four-item scale (CSQ-4) and a three-item scale evaluating patient satisfaction with the information provided about ADHD. In addition, four patient-reported outcome measures (PROMs) were applied: one scale to assess self-efficacy, two to assess ADHD symptoms, and one for quality of life. The statistical analysis applied intention-to-treat (ITT) and per-protocol (PP) analyses.

**Results:**

In total, 56 outpatients were divided into the IG (*n* = 30) or CG (*n* = 26), and the recruitment rate was 93.3%, with a dropout rate of 16%. The attendance rate was 92%, and the retention rates for the IG and CG were 56.6% and 76.9%, respectively. Concerning intervention satisfaction, the ITT analysis conducted using a linear mixed model revealed statistical improvements in satisfaction, with the scale measuring satisfaction with the information and the CSQ-4 scale. In addition, caregivers expressed high satisfaction with the programme, as measured by the CSQ-4 scale. Regarding PROMs, the ITT and PP analyses yielded mixed findings.

**Conclusion:**

Preliminary evidence indicates that a two-session, patient-centred, peer-cofacilitated psychoeducational programme is feasible and well-received, with high ratings for satisfaction from outpatients and caregivers.

**Cliniclatrials.gov Identifier:**

NCT03547843, 27/01/2022.

**Supplementary Information:**

The online version contains supplementary material available at 10.1186/s12888-025-06570-4.

## Background

Attention deficit hyperactivity disorder (ADHD) is a neurodevelopmental mental health condition [1] affecting functional impairments in academic performance, occupational performance, and relationships [2, 3]. Recent studies among adults with ADHD have suggested that patients want to understand their condition [4, 5]. However, behavioural problems, such as avoidance behaviour, failure orientation, and reduced self-efficacy [6, 7], create barriers to self-management, suggesting that psychoeducational interventions and support may be required.

A comprehensive treatment approach that addresses ADHD symptoms while empowering patients to participate in their treatment can be beneficial. This approach can be achieved by adopting a person-centred approach (i.e. integrating patients’ preferences, needs and values as treatment input) [8, 9]. Thus, ADHD symptoms can be managed in a way that considers the patient as a whole [1]. Notably, a person-centred approach fosters collaborative relationships between healthcare professionals, caregivers, and patients [10], empowering individuals to take an active role in their treatment [11]. This collaboration is critical for adults newly diagnosed with ADHD, who may struggle with many challenges [12].

Guidelines [13] and research [9] have underscored the benefits of a person-centred approach in mental health settings. To enhance adherence and treatment satisfaction, Khosravi, Azar and Izadi [9] advocated educating patients about their conditions and the available treatment options. The importance of encouraging active patient involvement and cooperation to foster a sense of ownership and empowerment has also been emphasised [9]. Although research findings on the clinical influence of person-centred interventions vary [14], studies in community mental health settings suggest promising outcomes, such as improved service delivery [15], increased knowledge of treatment preferences [16], greater patient satisfaction [17] and better mental health outcomes [18]. However, clinical studies from community mental health centres (CMHCs) are scarce and may not address the needs of adults newly diagnosed with ADHD.

Service users, patients, caregivers and peer support workers are increasingly involved in educational and training programmes [19]. For instance, Moreno et al. (2016) [20] found 64 resources, including guidelines, books, websites and podcasts, describing participation from user representatives in developing interventions. Other studies have described the value of collaboration between user representatives and health personnel when developing educational group interventions for patients with chronic conditions [21, 22]and serious mental health illnesses [15, 16, 23]. In Norway, legislation concerning patient education mandates the inclusion of expert patients, caregivers and service users [24]. This cocreation approach emphasises the active participation of service users, mental health organisations, and mental health professionals [25]. This approach involves individuals with the lived experience of mental health problems cofacilitating group sessions alongside professionals. Further, this approach is based on the principle of equality between peer or expert patients and healthcare professionals in planning and delivering educational group interventions [24, 26]. These programmes focus on peer support by encouraging group work and mutual support, enabling individuals to draw on the experiences of others [27].

Evidence demonstrates that peer-cofacilitated group programmes at the CMHC resulted in elevated patient activation [28], increased patient knowledge about treatment options [16, 29] and reduced dropout rates [18]. However, despite the growing interest in user involvement and patient education, a gap remains in research focusing on adults with ADHD and their families. For instance, Pedersen et al. [30] conducted a scoping review, finding that adults with ADHD have cofacilitated lectures about the diagnosis in psychoeducational programmes, such as the PEGASUS trial. Studies evaluating PEGASUS have demonstrated the effectiveness of structured group programmes for patients with ADHD and their significant others, highlighting improvements in ADHD knowledge and life satisfaction [31–33]. Skliarova et al. (2024) explored the feasibility and acceptability of a psychoeducational programme cofacilitated by users for adults newly diagnosed with ADHD [29]. This proof-of-concept RCT, with patients from a Norwegian CMHC, found that intervention group (IG) participants reported higher satisfaction with the information than the control group (CG). While the programme has promise, further research is necessary to confirm its effectiveness.

Moreover, only two studies involved family members [31, 33]. One study reported a significant decrease in caregiver burdens [33], although no changes were observed from the baseline to post-psychoeducation. In the second study [31], Hirvikoski et al. found that the PEGASUS psychoeducational programme benefitted adults with ADHD and provided support and education to their caregivers. However, no effects were reported on global life satisfaction or depression symptoms. Notably, neither of these studies evaluated satisfaction and acceptability using validated approaches. These findings highlight a gap in patient and family involvement in the design of psychoeducation programmes for adults with ADHD and also indicate the need for studies exploring the effects of educational programmes on client satisfaction.

We developed a psychoeducational group programme for adults diagnosed with ADHD to address these needs and promote the principles of person-centred care. The programme was developed with help from individuals with lived experience and healthcare providers. The programme comprises two sessions cofacilitated by peers, focusing on encouraging active outpatient participation, information provision and support via shared experiences and psychoeducation. The objectives are to investigate the feasibility of this group-based programme for adults with ADHD and assess acceptability by measuring outpatient and caregiver satisfaction in the programme. In addition, the final objective is to explore the potential benefits of this psychoeducational programme for adults diagnosed with ADHD to understand its effects on patient-reported outcomes related to self-efficacy, quality of life (QoL), and ADHD symptoms.

## Methods

### Study design

This pilot study employed a two-arm RCT design to evaluate a patient-centred education programme. The study involved a psychoeducational programme group (IG) and CG. Both groups received treatment as usual (TAU), but the IG also received a psychoeducational programme.

### Psychoeducational programme development and theoretical framework

The peer-cofacilitated psychoeducational programme was developed using the principles of patient-centred care, emphasising respect for individual preferences and collaboration and empowering the patient to make treatment decisions. This approach provides a theoretical framework for delivering personalised and collaborative care. In mental health contexts, this approach is particularly relevant when patients newly diagnosed with ADHD must navigate long-term challenges concerning treatment options and managing their condition. This intervention also draws on Bandura’s social learning theory, highlighting the importance of self-efficacy, observational learning and modelling [34]. This self-efficacy underscores the roles of knowledge and agency in enabling individuals to take control of their health. This collaborative approach reflects evidence from the literature highlighting the benefits of including peer education in enhancing knowledge retention and patient activation [15, 35].

This programme promotes collaboration aligned with patient-centred approaches by directly involving user representatives in teaching, unlike traditional psychoeducational programmes that providers lead. By involving caregivers and user representatives throughout the delivery process, the intervention aimed to foster an inclusive environment, empowering participants to become active partners in managing their treatment. The programme was coproduced through active collaboration with caregivers and user representatives from two user-led organisations in Norway: ADHD Norge and Vårres. Coproduction ensures that the programme addresses the lived experiences and practical needs of individuals affected by ADHD. Two caregivers from ADHD Norge played central roles in the development and delivery phases, contributing their insight to customising the programme to the needs of patients newly diagnosed with ADHD and their families. During the programme delivery, the user representative led one lecture and participated in discussions, answering questions and providing information on self-help groups that ADHD organisations provide. In addition, the representative was involved in the research process by discussing the outcomes and preparing supplemental materials.

**Fig. 1:**
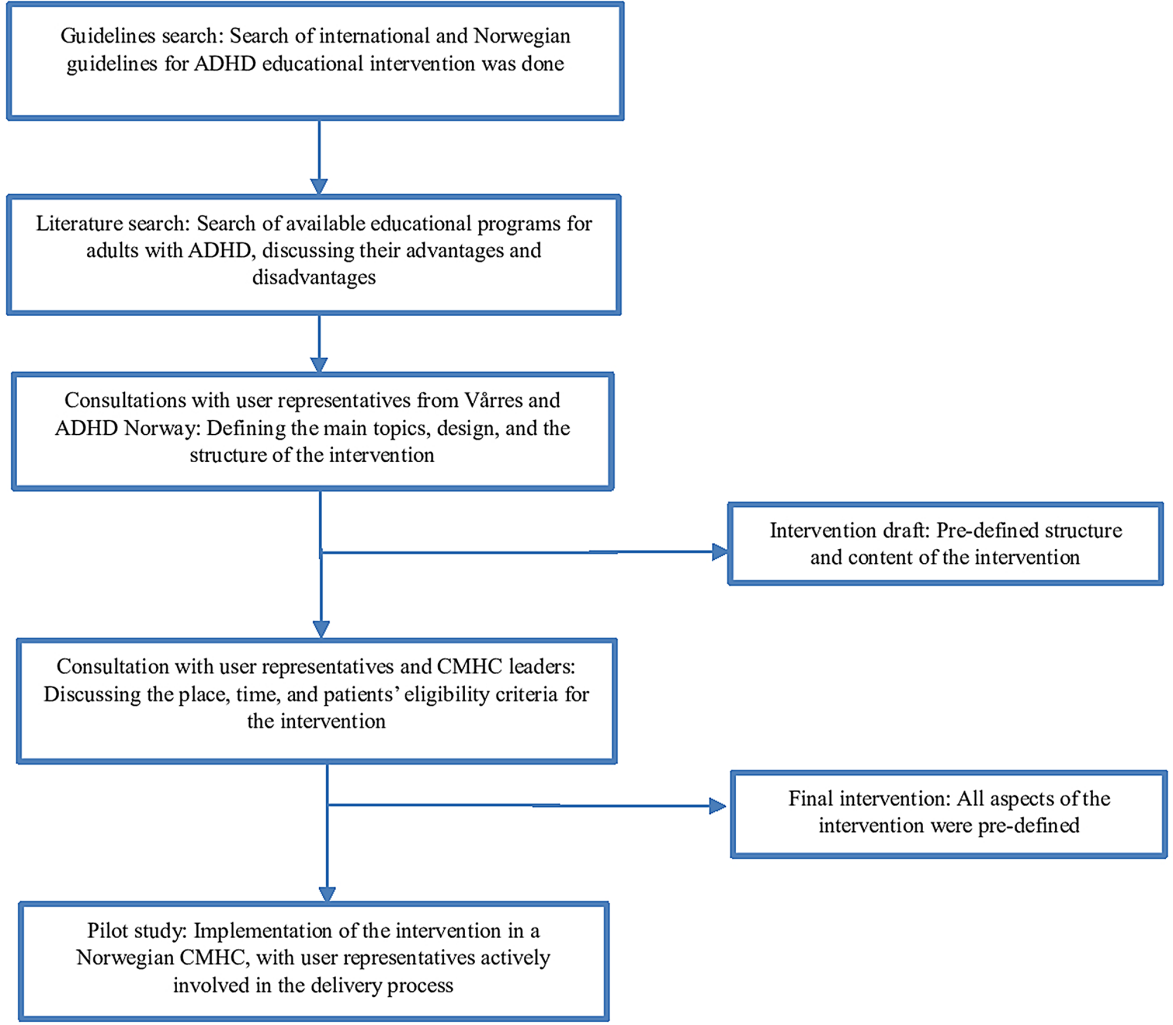
The process of developing the peer-cofacilitated psychoeducational programme

Figure 1 presents the process of developing the peer-cofacilitated psychoeducational programme. In this article, the intervention is delineated per the Template for Intervention Description and Replication (TIDieR) guidelines [36] (Table 1), whereas the RCT adheres to the Consolidated Standards of Reporting Trials for randomised pilot and feasibility trials [37] (Additional file 1).

**Table 1:** Overview of the peer cofacilitated intervention according to TIDieR criteria

TIDieR criteria	Description
Brief name	1. Peer Cofacilitated Psychoeducational Group Programme for Adults with ADHD
Why	2. To evaluate the feasibility, patient satisfaction, and effects on PROMs of a two-session peer cofacilitated educational group programme conducted in outpatient clinics in Norway for adults diagnosed with ADHD.
What	3. Materials: The intervention was conducted using PowerPoint presentations by group leaders. Participants received informative booklets about the study and information about available ADHD programmes/self-help organizations. 4. Procedure: Two educational sessions. The sessions were planned with time for group discussions to encourage patients to share their experiences and feelings about how ADHD symptoms have affected them, which should foster peer support. The topics of the sessions were as follows: Day 1 Introduction, presentation, and expectations; What is ADHD? Understanding its challenges; How to cope with ADHD; Further treatment possibilities (both pharmacological and nonpharmacological). Day 2 Economy, patient rights, and legal rights concerning treatment; Self-help and how to proceed with peer support and sharing experiences; Summary and closing session.
Provider	5. The educational programme was facilitated by a group leader (mental health nurse) and a user representative (caregiver). They were present throughout the sessions. Other educators were a psychiatrist and social workers with expertise in ADHD who provided didactic information supported by visual slides. The caregiver (user representative) was a role model, offering supplementary information and posing pertinent questions. The sessions focusing on patients’ rights were cofacilitated by user representatives and a social worker in such a way that the user representative asked questions and supplied relevant information.
How	6. The programme was delivered through in-person group sessions.
Where	7. Three outpatient clinics that were part of a CMHC in Norway.
When and how much	8. The cofacilitated group intervention was provided through two afternoon sessions running for 120 min for two consecutive weeks. Each session was divided into four sessions running for 20 min each, with short breaks to accommodate attention spans.
Tailoring	9. The intervention was developed for newly diagnosed ADHD patients and did not need to be personalized or adapted.
Modification	10. Minor modifications were made to the content of the PowerPoint presentations and patients’ booklets.

Note ADHD: Attention deficit/hyperactivity disorder, TIDieR: Template for Intervention Description and Replication guidelines

In terms of structure, during both sessions, shared learning and the value of the lived experience were emphasised, providing participants with relatable insight. In addition, outpatients were encouraged to participate in their treatment through group discussions cofacilitated by a user representative, fostering mutual respect and the sharing of diverse perspectives. Furthermore, the information on self-help groups and supplemental materials was delivered to outpatients to empower them to self-manage their condition.

### Intervention and control groups

#### Intervention group

The IG received a person-centred educational programme consisting of two sessions given in two consecutive weeks. Each session lasted 2 h and was divided into four segments, each lasting 20 min, with short breaks between to accommodate possible attention difficulties. Table 2 presents the intervention content. The intervention focused on self-managed education and self-efficacy, delivering information about the diagnosis and treatment options to help outpatients feel more in control of their condition and more confident in their ability to manage ADHD.

**Table 2:** Content of the educational programme

Day 1	Responsible
Introduction, presentation and expectations	Course leaders (a mental health nurse and an expert patient or user representative)
What is ADHD? Understanding its challenges based on a biopsychosocial approach	A nurse, psychiatrist or psychologist with ADHD expertise and a physical therapist
How to cope with ADHD	An expert patient and mental health nurse or psychologist with ADHD expertise
Treatment options (pharmacological and nonpharmacological)	A psychiatrist specialised in ADHD
Day 2	Responsible
Economy, patient’s rights and legal rights concerning treatment options	A social worker and a user representative
Sharing experiences, self-help and how to proceed with peercofacilitated groups	A user representative from ADHD Norway
Summary and closing session	Course leaders (a mental health nurse and an expert patient or user representative)

Note ADHD: attention deficit hyperactivity disorder

The educational programme was facilitated by a mental health nurse and a user representative, who were present for the sessions. Educators with backgrounds as psychiatrists, psychologists, physical therapists and social workers with expertise in ADHD provided information supported by visual slides. Outpatients received informational brochures and written materials. Structured group discussions were encouraged following the presentations to facilitate peer support and sharing experiences related to ADHD symptoms.

### Control group

At the participating clinics, participants in the CG received TAU, reflecting the standard care protocol for adults newly diagnosed with ADHD. Licenced therapists provided TAU, including individualised clinical counselling sessions tailored to each patient’s needs and circumstances. In addition to TAU, each participant in the CG attended a 1-h informational session designed to provide a basic understanding of ADHD. This session was delivered by a researcher or staff member not directly involved in the group intervention. The session covered core topics, such as the nature of ADHD, common challenges faced by individuals with the condition and an overview of the available treatment options, including medication and group treatment.

However, unlike the IG, the informational session did not involve peer coleadership, group discussions, family education or supplemental resources. Moreover, it was not tailored to incorporate patient-centred care principles, such as active patient engagement. In addition, the distinction between the CG and IG is the interaction and peer support the user representatives provide. The CG TAU approach focused on professional-led guidance and general ADHD education, whereas the IG emphasised collaborative learning, peer-cofacilitated education and empowerment. Those in the CG were not informed of the possibility of inviting their caregivers to participate in their group intervention. Caregivers were defined as parents, partners, or other close family members such as siblings.

### Recruitment

Patients newly diagnosed with ADHD were recruited at a specialised Norwegian CMHC. Clinicians in the outpatient units received written details about the study. The clinicians were requested to inform eligible outpatients and distribute information letters to them. Before recruitment, all participants underwent the first consultation in an outpatient unit and received their psychiatrists’ recommendations and prescriptions for medical treatment (if necessary).

Inclusion criteria were having a confirmed ADHD diagnosis, being at least 18 years old, and having the ability to speak a Scandinavian language. Exclusion criteria were psychosis and severe learning difficulties, as assessed by the patients’ therapists. Outpatients were not invited to participate if they could not provide informed consent. The ethical committee approved the study (2017/2405 REK).

A researcher or clinician (group therapists) screened all referrals for eligibility. Patients interested in participating were invited to the first meeting, where outpatients received written and verbal information on the study. In addition, before providing written informed consent, outpatients received oral information about their right to withdraw from the RCT at any time.

### Randomisation

Randomisation procedures were designed and implemented to ensure methodological rigour and reproducibility. Following the informed consent, participants completed baseline questionnaires to collect demographic information and baseline measures of the critical outcomes. This step ensured that randomisation was conducted only after obtaining the necessary data to compare the groups at the baseline. Independent computer-assisted software employed a block randomisation procedure to assign participants randomly to the IG and CG. This approach was selected to maintain balanced group sizes throughout recruitment, minimising the risk of unequal group allocation. A Norwegian University of Science and Technology research unit in Norway conducted this randomisation process independently. Randomisation was delegated to this independent research unit to minimise potential biases and safeguard the integrity of the group allocation process. The block size and randomisation sequence were predetermined and concealed from all study personnel in the recruitment and data collection processes. This measure aimed to prevent selection bias and enhance the reproducibility of the study. After randomisation at the CMHC, outpatients were promptly informed of their group assignments by a researcher or clinician not directly involved in the intervention delivery. Recruitment was completed after screening 60 participants, of whom 56 were eligible and randomised into a group.

Participants in the IG were informed about inviting their caregivers to participate in the group intervention. They were given written information about the date and location of the group intervention. This written information was also provided to eligible caregivers, who were informed about the anonymity of the study to protect confidentiality and encourage open participation.

Randomisation occurred between April 2018 and January 2019. The educational group programmes were held once per semester. The first course was conducted on May 29 and June 5, 2018. The second course was on September 4 and 11, 2018, and the final course was held on March 13 and 30, 2019. The periods between randomisation (T_0_) and the post-intervention evaluation (T_1_) varied, with a mean of 41.22 days (*SD* = 21.27) for participants from the IG. For participants in the CG, the period between T_0_ and T_1_ was *M* = 64.81 (*SD* = 19.95) days.

### Sample size

This pilot RCT study investigated the feasibility of a psychoeducational group programme; therefore, the formal sample size was not calculated in advance. Consequently, the minimum sample size was set to 12 participants per arm based on the recommended sample size for pilot and feasibility studies [38].

### Data collection and outcome measures

Data were collected from both groups at two points. First, T_0_ marks the time before randomisation when an informational meeting was held, informing participants about the intervention. At this time, outpatients completed the paper-based baseline questionnaires. Second, T_1_ is at the end of the study during post-intervention for the IG and the final assessment for the CG. Data at T_1_ were collected after the psychoeducational group programme was completed. At each time point, measurements were taken for both groups to assess the degree of self-efficacy, ADHD symptoms, QoL and patient satisfaction with the information received. Data from caregivers who participated in the first educational sessions were collected only at T_1_. Caregiver satisfaction was measured via an anonymous paper-based questionnaire.

### Feasibility outcomes

Based on the 2022 guidelines by Teresi et al. [39], feasibility was evaluated across the following domains:

#### Consent to participate

(1) was established in advance, with feasibility set at 50% of eligible patients accepting participation. This threshold was based on previous studies and recommendations [40–42], suggesting that a 50% enrolment rate is a reasonable benchmark for feasibility in similar contexts.

(2) The adherence rate was defined as the percentage of sessions attended by participants and the number of participants who attended one or two in-person sessions. A previous study [33] reported an adherence rate of 50%, indicating that participants should attend at least half of the sessions. Other studies have reported adherence rates ranging from 79% [31] to 87% [43]. Thus, an attendance rate of at least 50% is a reasonable benchmark for this feasibility indicator.
(3) The dropout rate was defined as the percentage of participants who did not complete the intervention. The dropout rate varies from 5 to 40% [44], depending on study design, intervention and population. However, a previous study based on psychoeducation for the adult ADHD population set the limit of the dropout rate at 25% [33]. In line with this feasibility benchmark, the dropout rate in this study did not exceed 25%, aligning with the expectations set by prior research.
(4) Resources used. We collected data to describe the time and resources used by the professionals involved in conducting the intervention. This included detailed information of the hours spent by nurses, social workers, and psychiatrists, ensuring a comprehensive understanding of the resource allocation required for the intervention.

### Acceptability outcomes

The second objective was to assess acceptability by measuring the outpatient and caregiver satisfaction with the programme. For outpatients, acceptability was assessed based on two measures for satisfaction. The first assessed acceptability using the four-item Client Satisfaction Questionnaire (CSQ-4) [45]. This scale was previously employed as an acceptability indicator to evaluate patient satisfaction with a psychoeducational group programme [29]. Moreover, this scale has good psychometric properties [46] and was validated in an outpatient psychiatric centre in Norway [45]. The CSQ-4 was selected due to its proven reliability and validity in Norwegian contexts [46], making it an appropriate tool for assessing client satisfaction in this study.

The CSQ-4 comprises the four questions listed below (Table 3). Each CSQ-4 question was scored from 1 to 4, resulting in a mean score from 4 to 16, with high scores indicating excellent patient satisfaction.

**Table 3:** Satisfaction of outpatients and caregivers based on item responses on an item-by-item basis

CSQ-4 Items	Caregivers		IG patients		CG patients	
	M (SD)	*n* (%) satisfied	M(SD) IG	*n* (%) IG	M (SD) CG	*n* (%) CG
Item 1: Extent to which the programme meets participant needs	2.87 (0.640)	11 (73.3%)	2.94 (0.556)	14 (82.4%*)	2.47 (0.717)	8 (47.1%)
Item 2: Level of help provided by the programme in managing participant problems	2.93 (0.458)	13 (87.6%*)	3.12 (0.600)	15 (88.2%*)	2.88 (0.696)	12 (70.5%)
Item 3: Overall satisfaction with the programme	3.13 (0.352)	15 (100%*)	3.24 (0.664)	15 (88.2%*)	2.76 (0.752)	10 (58.8%)
Item 4: Likelihood of returning to the programme for future help	3.20 (0.676)	13 (86.7%*)	3.35 (0.492)	17 (100%*)	3.29 (0.469)	14 (82.4%*)

Note *n* (%): Number and percentage of participants classified as satisfied. CG: control group, CSQ-4: Four-item Client Satisfaction Questionnaire, IG: intervention group, *M*: mean, *SD*: standard deviation. Bold text* indicates satisfaction rates at or above the 75% threshold

The second measure to assess outpatient acceptability was patient satisfaction with the information about ADHD and treatment options (SIATS) [47], assessed at T_0_ and T_1_. The SIATS is a brief, self-rated scale that employs three items: measured participant perceptions of the information received regarding ADHD, treatment options, and pharmacological treatment. The three items were rated on a 5-point Likert scale, from 1 (“not satisfied”) to 4 (“very satisfied”), along with the option “I don’t know” (rated as 0). The maximum total score for the questionnaire was 12, indicating the highest level of satisfaction with the information received (Table 4).

**Table 4:** Patient satisfaction with the provided information

Items	IG, M (SD)	CG, M (SD)	*p*	95% CI
How do you perceive the information provided to you?				
Item 1: ADHD	3.27 (0.88)	2.30 (1.34)	0.002	[0.441, 1.857]
Item 2: treatment options	3.36 (0.75)	2.05 (1.39)	0.004	[0.393, 1.851]
Item 3: medications	3.07 (1.14)	2.37 (1.12)	0.009	[0.284, 1.859]

Note ADHD: attention deficit hyperactivity disorder, CI: confidence interval, CG: control group, IG: intervention group, M: mean, *SD*: standard deviation

For caregivers, acceptability was assessed using the CSQ-4. Focusing on caregiver satisfaction, the study emphasised the importance of the caregiver’s perspective in evaluating treatment success. This approach can lead to more patient-centred treatment and improved caregiver satisfaction. The caregivers completed this questionnaire at T_1_ to measure their satisfaction with the programme.

### Patient-reported outcomes related to preliminary efficacy

Patient-reported outcome measures (PROMs), including self-efficacy, ADHD symptoms and QoL, were the secondary outcome measures, emphasising the importance of the patient’s perspective in evaluating treatment success. Self-efficacy was predefined as a primary PROM. The General Self-Efficacy (GSE) Scale for adults with ADHD [48] was selected as the primary measure due to its proven reliability and validity in Norwegian contexts and its appropriate use in a previous RCT study [29]. Table 5 lists the characteristics of the PROMs and their internal consistency measures.

**Table 5:** Characteristics of outcome measures

PROMs		Description	No. of items	Range	Cronbach’s alpha
Self-efficacy	GSE-6-ADHD	GSE-6 comprises six items assessed using a 4-point Likert scale (1–4). The highest total score indicates the highest self-efficacy. GSE-6 is validated for adults diagnosed with ADHD.	6	6–24	0.867
ADHD symptoms	SCL‑9	SCL-9 comprises nine items assessed with a scale from 0 to 4; a high score indicates pronounced symptoms.	9	0–36	0.863
	ASRS	ASRS comprises 18 items distributed into two subscales: Questions 1–9 comprise the inattention subscale, and 10–18 comprise the impulsivity and hyperactivity subscale. Item scores range from 0 to 4, with a maximum overall score of 72, indicating severe symptoms.	18	0–72	0.825
Quality of life (QoL)	AAQoL	AAQoL comprises 29 questions across four subscales: life productivity, psychological health, relationships and life outlook. Responses are evaluated using a 5-point Likert scale. The overall score is converted to a 100-point scale, where a higher score signifies a better QoL.	29	0–100	0.908

Notes AAQoL: Adult ADHD Quality of Life Scale, ADHD: attention deficit hyperactivity disorder, ASRS: Adult ADHD Self-Reported Scale, GSE-6: Abridged Version of the General Self-Efficacy Scale for adults with ADHD, SCL-9: Hopkin’s Symptom-Checklist Scale

### Statistics

The IBM SPSS Statistics (v.29) software was employed for the statistical analysis. Descriptive statistics, such as the proportion, mean and standard deviation, were employed to summarise demographic data and scale scores. Group differences for continuous variables were assessed using independent sample *t*-tests or Mann–Whitney U tests. A chi-squared test of associations for categorical data or Fisher’s exact test was used. Cohen’s *d* was applied to assess the effect size between the two groups as follows: *d* = 0.2 (small effect size), *d* = 0.5 (medium effect size), and *d* ≥ 0.8 (large effect size) [49]. Furthermore, absolute numbers and percentage frequencies describe the feasibility indicators, including consent, adherence and dropout rates.

### For CSQ-4

First, the overall mean CSQ-4 score was calculated by summing the responses to all four questions. A score of 12 to 16 (out of 16 points) indicated high satisfaction, whereas scores below 12 indicated low satisfaction. Thus, a mean CSQ-4 score above 12 indicates that the programme is satisfactory.

Second, outpatient and caregiver satisfaction scores were evaluated on an item-by-item basis using the CSQ-4. For each item, if a patient scored 3 or 4 (on a scale from 1 to 4), the result was classified as indicating satisfaction with that aspect of the programme. Conversely, if patients scored an item as 1 or 2, they were considered dissatisfied with that area. A previous study [29] similarly used CSQ-4 as an acceptability indicator, considering the acceptability threshold to be 3 or 4. In the present study, when at least 75% of participants scored each of the CSQ-4 scale items at 3 or 4, this result met the threshold for considering this programme aspect satisfactory. In this study, the satisfaction classification threshold (percentage of participants rating their experience with each programme aspect as at least 3 or 4) was expected to exceed 75% for all items.

The intention-to-treat (ITT) and per-protocol (PP) analyses were conducted to measure the acceptability indicator and PROM scores. Mean differences for secondary outcomes at the time points were assessed via the linear mixed model analysis in SPSS. Time points and treatment groups (with ‘baseline’, ‘T_1_ for IG’ and ‘T_1_ for CG’) were treated as fixed effects; participant IDs were considered a random effect. The significance level was set at *p* <.05.

## Results

### Recruitment and feasibility

**Fig. 2:**
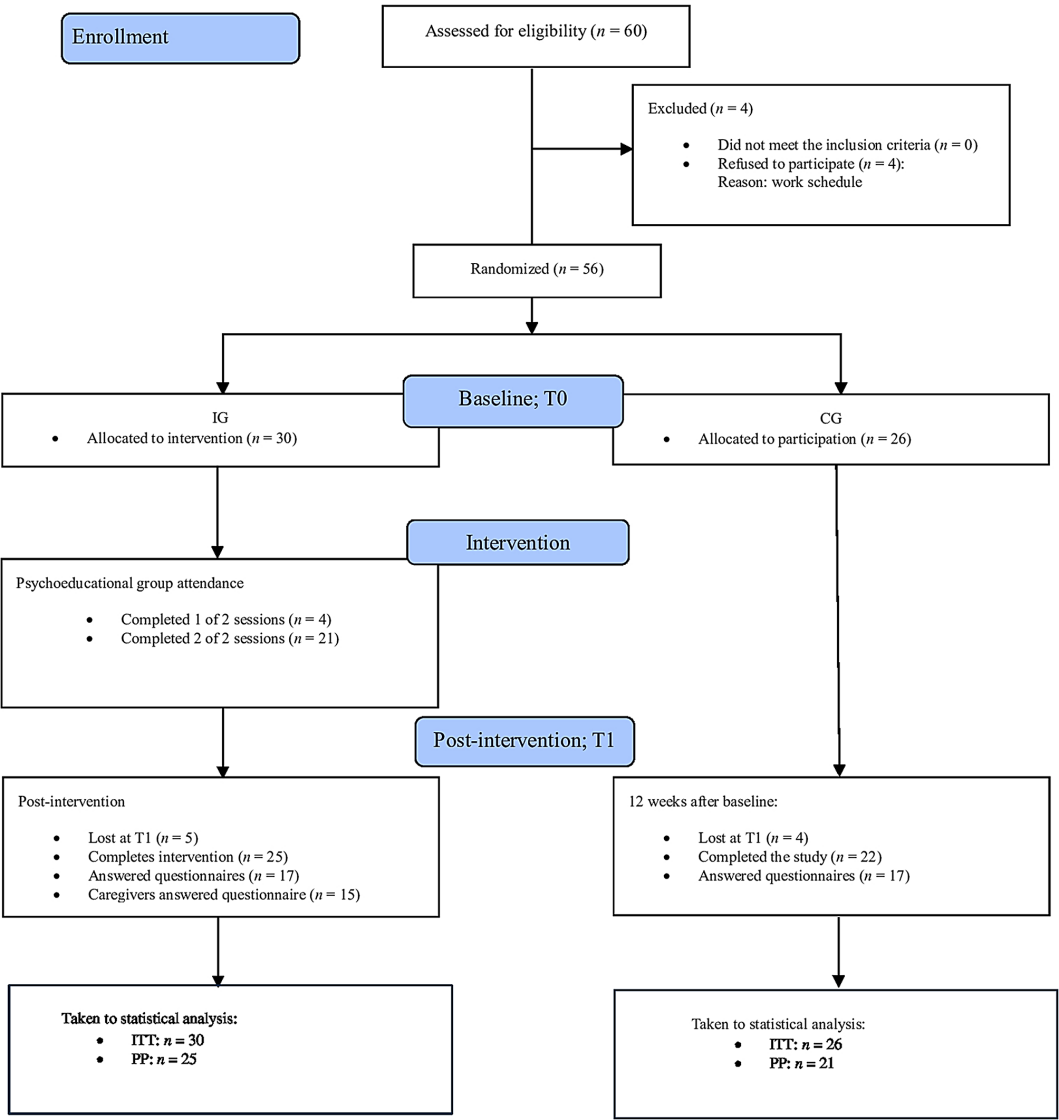
Flow of participants through the peer co-led educational interventions study. Note: CG: Control group, IG: Intervention group, ITT: Intention-to-treat analysis, PP: Per-protocol analysis

In this study, 60 outpatients were invited to participate, and 56 consented. The mean age of the sample was 33.7 years (SD = 9.7), ranging from 18 to 53. The mean age was 35.3 (SD = 8.3) in the IG and 31.9 (SD = 10.9) in the CG. Table 6 presents the demographic characteristics of the participants by group and the differences in the total scores between the groups at baseline. Figure 2 illustrates the recruitment process (CONSORT flow diagram).

**Table 6:** Demographic and baseline characteristics

Characteristics	Total sample (*N* = 56)	IG (*n* = 30)	CG (*n* = 26)	*p*-value
**Gender**, ***n*(%)**				0.170
Male	21 (37.5%)	14 (46.7%)	7 (26.9%)	
Female	35 (62.5%)	16 (53.3%)	19 (73.1%)	
**Relationship**, ***n*** **(%)**				0.597
Single	23 (41.1%)	11 (36.7%)	12 (46.2%)	
Married	8 (14.3%)	6 (20.0%)	2 (7.7%)	
Live with a partner	20 (35.7%)	10 (33.3%)	10 (38.2%)	
Divorced	5 (8.9%)	3 (10.0%)	2 (7.7%)	
**Education**, ***n*(%)**				0.194
Primary/secondary school	12 (21.4%)	4 (13.3%)	8 (30.8%)	
Post-secondary school	31 (55.4%)	17 (56.7%)	14 (53.8%)	
University	13 (23.2%)	9 (30.0%)	4 (15.4%)	
**Employment**, ***n*** **(%)**				0.109
Student	8 (14.3%)	2 (6.7%)	6 (23.1%)	
Employed	24 (42.9%)	16 (53.3%)	8 (30.8%)	
Partially employed (50%)	5 (8.9%)	4 (13.2%)	1 (3.8%)	
Disabled	3 (5.4%)	2 (6.7%)	1 (3.8%)	
Unemployed	16 (28.6%)	6 (20.0%)	10 (38.5%)	
**ADHD medication**, ***n*(%)**				1.00
Yes	31 (55.4%)	17 (56.7%)	14 (53.8%)	
No	25 (44.6%)	13 (43.3%)	12 (46.2%)	

Note ADHD: attention deficit hyperactivity disorder, CG: control group, IG: intervention group, *M*: mean, *n*: number of participants, *SD*: standard deviation

### Feasibility

The study met the criteria to assess the feasibility of the intervention. The recruitment rate was 93.3%, exceeding the predefined threshold for acceptable enrolment set at 50%. Regarding the adherence rate, defined as the percentage of sessions attended by participants and number of participants attending one or two in-person sessions, the threshold for acceptable feasibility was set at 50%. The attendance rate in this study was 92%, significantly exceeding the predefined threshold.

Regarding the dropout rate, defined as the percentage of participants not completing the intervention, a threshold for acceptable feasibility was set not to exceed 25%. In this study, nine participants dropped out, resulting in a dropout rate of 16%. This rate is well below the predefined threshold, indicating acceptable feasibility. In the CG, 15.4% (4 out of 26) dropped out, whereas in the IG, the dropout rate was 16.6% (5 out of 30). Of the nine participants who dropped out, five cited personal reasons (one had a busy schedule, two were sick and two needed to prioritise work).

Regarding the resources to conduct the intervention, two courses were conducted so that all participants in the IG received the group intervention. Concerning the time spent by those delivering the intervention (receiving a salary) for each course, a nurse spent 2 h each on two sessions (4 h). The social worker spent 60 min (total 2 h), and the psychiatrist spent 90 min for each course (total 3 h), for the two courses in the IG.

For data collection, at T_1_, 17 of the IG participants completed questionnaires (56.6%), and 17 CG participants completed questionnaires (76.9%). All caregivers who participated in the sessions (*n* = 15) completed the CSQ-4 questionnaire at T_1_.

### Acceptability

The study met the criteria to assess the acceptability of the intervention measured using two measures for satisfaction among outpatients. The first acceptability measure was assessed using the CSQ-4. The overall mean score for the IG was 12.68 (Table 7). Since this score exceeds 12, the threshold of high satisfaction was met for patients participating in the programme. In contrast, the CG had an overall mean satisfaction score of 11.69 (Table 7), which did not meet the high satisfaction threshold. Using the CSQ-4 on an item-by-item basis, the IG met the 75% satisfaction threshold for all items (Table 3). In contrast, the CG met the 75% satisfaction threshold for only one item.

**Table 7:** Intention-to-treat and per-protocol analyses of acceptability outcomes using a linear mixed model

Acceptability outcome	Mean at T_0_		Model-based Mean at T_1_ Post-intervention					
	IG	CG	IG	CG	Diff	*p*	95% CI	Cohen’s d
Intention-to-treat analyses								
SIATS: total mean score	6.379	5.666	9.251	6.470	2.781	0.001	[1.171, 4.392]	1.404 [0.589, 2.200]
CSQ-4: total mean score	12.069	11.693	12.450	11.571	0.879	0.051	[-0.006, 1.765]	0.618 [-0.076, 1.302]
**Per-protocol analysis**								
SIATS: total mean score	5.920	5.750	9.229	6.182	3.047	< 0.001	[1.430, 4.665]	1.340 [0.488, 2.169]
CSQ-4: total mean score	12.040	11.619	12.679	11.695	0.984	0.037	[0.064, 1.904]	0.822 [0.091, 1.540]

Note CG: control group, CI: confidence interval, CSQ: Client Satisfaction Questionnaire, IG: intervention group, M: mean, SD: standard deviation, SIATS: Satisfaction with the received Information about ADHD and Treatment Scale

Moreover, the PP analysis via a linear mixed model revealed statistically significant differences between the groups regarding CSQ-4 (*p* = .037, Table 7). In the ITT analysis, CSQ-4 displayed a medium positive effect size, whereas satisfaction with the information (SIATS) presented a large effect size (Cohen’s *d* = 1.404). Both acceptability measures (CSQ-4 and SIATS) indicated a positive large effect size in the PP analysis, with Cohen’s *d* at 0.822 and 1.340, respectively.

For the second outpatient acceptability measure SIATS (measured post-intervention), the IG reported significantly higher satisfaction with information on ADHD (*p* = .002), treatment options (*p* = .004) and medications (*p* = .009) than the CG (Table 4). The ITT analysis revealed statistically significant differences between groups (Table 7).

Acceptability was measured among caregivers using the CSQ-4. First, the overall mean CSQ-4 score across all items for caregivers was 12.13. This score exceeds 12; hence, the threshold of high satisfaction was met for caregivers participating in the programme. Second, considering the satisfaction levels on an item-by-item basis, three of the four items exceeded the 75% threshold to consider these programme aspects satisfactory. The highest satisfaction (100%) was observed for the overall satisfaction with the programme. Moreover, all caregivers were satisfied with the programme, and 86.7% of participants indicated that they would return to the programme if they needed it. Table 3 presents more detailed information about caregiver and patient satisfaction.

### PROMs for preliminary efficacy

The ITT analysis revealed significant differences between IG and CG in the psychological health subscale of QoL (*mean difference* = 10.838; *p* = .034, CI [0.850, 20.825]). However, other PROMs did not demonstrate statistically significant differences between the groups. The PP analysis, which only included participants who adhered to the intervention protocol, revealed mixed results. Tables 8 and 9 detail the information on the effects observed in the ITT and PP analyses. Effect sizes measured using Cohen’s *d* were as follows: The Abridged Version of the GSE for adults with ADHD (GSE-6) displayed large effect sizes, and the Hopkin’s Symptom-Checklist Scale (SCL-9) total score, Adult ADHD Self-Reported Scale (ASRS), and Adult ADHD Quality of Life Scale (AAQoL) revealed small-to-medium effect sizes in different subscales.

**Table 8:** Intention-to-treat analysis using a linear mixed model

PROMs	Model-based Mean at T0	Model-based Mean at T1 Post-intervention					
	Baseline	IG	CG	Diff	*p*-value	95% CI	Cohen’s d
GSE-6; total mean score	15.929	16.737	15.809	0.927	0.300	− 0.855; 2.709	0.873 [0.138,1.595]
SCL-9; total mean score	20.327	16.797	18.853	-2.056	0.242	-5.556; 1.444	− 0.149 [- 0.843,0.547]
ASRS; total mean score	48.091	47.586	47.673	− 0.087	0.945	-2.665; 2.490	− 0.325 [-1.022,0.377]
ASRS; subscale inattention	25.722	26.423	25.781	0.642	0.506	-1.280; 2.564	− 0.497 [-1.186,0.201]
ASRS; subscale hyperactivity	22.393	20.280	22.249	-1.970	0.059	-4.019; 0.080	− 0.042 [- 0.736,0.653]
AAQoL; total mean score	45.418	51.449	46.334	5.115	0.122	-1.434; 11.665	0.267 [- 0.443,0.972]
AAQoL; subscale life productivity	42.873	47.970	40.954	7.016	0.086	-1.035; 15.067	0.277 [- 0.433,0.983]
AAQoL; subscale psychological health	43.526	53.457	42.619	10.838	0.034	0.850; 20.825	0.344 [- 0.358,1.041]
AAQoL; subscale relationship	50.091	53.683	53.692	− 0.009	0.998	-8.250; 8.232	0.034 [- 0.660,0.728]
AAQoL; subscale life outlook	46.000	51.755	47.580	4.174	0.310	-4.020; 12.369	0.298 [-. 413,1.004]

Note AAQoL: Adult ADHD Quality of Life scale, ASRS: Adult ADHD Self-Reported Scale, CG: Control group, GSE-6: Abridged Version of the General Self-Efficacy scale for adults with ADHD, IG: Intervention group, SCL-9: Symptom-Checklist scale

**Table 9:** Linear mixed model per-protocol analysis

PROMs	Model-based Mean at T0	Model-based Mean at T1; Post-intervention		Model-based difference between groups at T2		
	Baseline	IG	CG	Diff	*p*-value	95% CI
GSE-6; total score	15.830	16.629	15.677	0.951	0.274	− 0.780, 2.683
SCL-9; total mean score	20.087	16.647	19.169	-2.522	0.173	-6.203, 1.158
ASRS; total mean score	48.809	45.131	49.622	-1.788	0.017	-8.137, − 0.844
ASRS; subscale inattention	26.340	24.905	27.223	-2.318	0.048	-4.611, − 0.025
ASRS; subscale hyperactivity	22.468	20.509	22.511	-2.003	0.075	-4.224, 0.218
AAQoL; total mean score	44.240	49.791	44.686	5.104	0.135	-1.666, 11.875
AAQoL; subscale life productivity	41.618	46.245	39.077	7.168	0.088	-1.115, 15.450
AAQoL; subscale psychological health	43.3151	51.872	41.025	10.847	0.035	0.790, 2.709
AAQoL; subscale relationship	48.617	51.296	51.234	0.062	0.998	-8.461, 8.586
AAQoL; subscale life outlook	45.537	52.034	47.141	4.893	0.262	-3.799, 13.585

Note AAQoL: Adult ADHD Quality of Life scale, ASRS: Adult ADHD Self-Reported Scale, CG: Control group, GSE-6: Abridged Version of the General Self-Efficacy scale for adults with ADHD, IG: Intervention group, SCL-9: Symptom-Checklist scale

## Discussion

Patient-centred educational interventions are crucial for promoting patient engagement, enhancing a patient’s QoL and increasing patient satisfaction. This pilot RCT study found that a peer-cofacilitated, person-centred educational programme was feasible and acceptable for adults diagnosed with ADHD.

The recruitment rate was higher than in other studies on the ADHD adult population [31, 50–52] at 78.6–95.8%. The same trend occurs in user-representative studies, where the acceptance rate ranges from 91% [16] to 93.8% [29]. Only four of the 60 outpatients chose not to enrol in the study. Patients gave several reasons for not participating, such as work commitments, difficulty taking a day off, and distance from the CMHC. Compared with other studies [16, 51, 52], where enrolment rates varied between 26% [52] and 52% [51], the current study had a high enrolment rate. This finding reflects the willingness to participate in the educational programme involving user representatives.

The retention rate post-intervention was comparable to that in previous studies [44, 46–49, 51, 52], reporting rates ranging from 66.6% [52] to 100% [49]. Furthermore, the attendance rates were higher than in similar studies reporting attendance rates of 82% [29], 86% [31] and 87% [43]. Hirvikoski et al. conducted studies based on the ‘PEGASUS’ psychoeducational programme in 2015 and 2017, setting an acceptable attendance level at 50% [31, 33]. In this study, 92% of the sessions were attended. This high attendance rate can be attributed to the inclusion of newly diagnosed patients who were likely highly motivated to participate. The programme was scheduled outside of work hours and comprised only two sessions, which may have contributed to the higher attendance than other educational programmes [31, 43, 53].

The dropout rates were 16.6% and 15.4% in IG and CG, respectively. These rates are comparable to those reported in other studies focused on group educational programmes for ADHD adults [33, 51, 54]. For instance, some studies conducted on adults with ADHD [53, 55] have documented a higher dropout rate of 27%; however, these studies were based on individual psychoeducation. This finding suggests that group educational programmes may be more effective in retaining participants, likely due to the supportive group environment [56]. Group programmes also offer a cost-effective alternative for the healthcare system, making them a viable option for routine practice [57]. The lower financial burden, combined with the lower dropout rates, supports the implementation of group psychoeducation as a standard approach for adults with ADHD. Participants who dropped out cited a ‘busy schedule’ and ‘work priority’. These factors suggest the need for flexible programme delivery. In addition, information about ADHD could be provided through digital resources to accommodate varying schedules, or group sessions could be scheduled at times that better align with participant availability.

Regarding acceptability measured with the CSQ-4, the results are comparable with those in previous research [29], where at least 75% of participants indicated satisfaction with the educational programme. In addition, the overall score for satisfaction with the programme in this study exceeds the threshold of 12, aligning with a previous study [29], where the mean CSQ-4 score was 12.83. All IG participants demonstrated a willingness to participate in similar programmes in the future, indicating the need to obtain more information on the disorder and to extend the psychoeducational programme for patients.

Another critical acceptability outcome was the satisfaction with the information, measured using the SIATS self-report scale, revealing significant differences between the IG and CG. This finding aligns with a previous RCT [29]. However, although this finding is preliminary and few clinical studies have reported satisfaction with the received information as an acceptability outcome, the significant effect size of the SIATS in this study underscores the clinical importance of psychoeducational interventions, consistent with earlier research [29]. Moreover, satisfaction with the received information predicts overall satisfaction with mental healthcare services [47].

However, the findings are limited because the SIATS has not been validated and is not a standardised acceptability measure. This finding relies on self-reported measures, which can be biased. For instance, participants might have reported their satisfaction inaccurately due to social desirability or recall bias. Future studies evaluating psychoeducational group programmes should include additional acceptability measures beyond self-reported data to address these limitations. Furthermore, patient-reported scales that measure satisfaction with information and psychoeducational programmes for adults with ADHD must be validated and standardised. Future validation studies should facilitate more accurate comparisons.

Caregiver acceptability was anonymously measured using the CSQ-4, demonstrating high satisfaction with the programme. The results align with previous studies [31, 33], where caregivers reported good treatment satisfaction [33]. In one of these studies [33], individuals with ADHD had higher scores than their caregivers and reported their willingness to participate in similar programmes in the future. This study yielded similar results in those participants with ADHD who reported a willingness to return to the programme.

However, fewer caregivers than outpatients reported their willingness to return to the programme. One possible explanation could be that caregivers were invited to only one of the two sessions. Nevertheless, no significant group differences were found in client satisfaction when comparing outpatients and caregivers in the intervention. This outcome aligns with findings in previous studies [33]. Thus, the psychoeducational programme is equally suitable and meets the needs of outpatients and their caregivers. Participation in such programmes with caregivers can contribute to satisfaction with the provided information [58], but more studies are needed to replicate these findings. Future studies should also measure other satisfaction aspects and contribute to more effective interaction between patients with ADHD and their caregivers [59].

This pilot RCT study highlights the importance of PROMs by focusing on the patient’s perspective in evaluating preliminary efficacy, with self-efficacy predefined as the primary outcome. However, the results did not indicate significant improvement in self-efficacy levels. In contrast, other studies have found that peer-cofacilitated psychoeducational programmes can significantly enhance self-efficacy among adults with mental health problems [49, 60–62] and children with ADHD [63]. One possible explanation for the findings is the duration of the programme. A brief, two-week intervention may not be sufficient to develop and strengthen self-management skills.

Although the results did not indicate significant improvement in self-efficacy levels, the effect size, measured by Cohen’s *d*, was large, suggesting a substantial difference in mean scores [49] and clinical significance [60]. Nevertheless, the discrepancy between the lack of statistical significance and the large effect size could be attributed to the low sample size, which was likely insufficient to achieve statistical significance [48, 58]. Another explanation and limitation is that the study applied a global and trait-like measure of self-efficacy. Future studies should use a domain-specific measurement of self-efficacy to investigate potential changes in ADHD-related self-efficacy beliefs. Exploring whether a domain-specific self-efficacy scale can improve this outcome among adults with ADHD would be a valuable direction for future research.

The PP analysis revealed significant improvement in ADHD symptoms, consistent with previous studies investigating the effects of psychoeducational group programmes. For instance, Hirvikoski [31] and Hartung [43] also reported improvements in ADHD-related impairment. Although some RCTs with follow-ups have reported improvement in adult ADHD symptoms [54, 55], others have not [43, 50, 51, 53]. These mixed results could be due to differences in interventions, such as the duration, delivery method (digital or in-person), medications or duration of the follow-up period. Hence, additional studies addressing these factors are necessary.

In one subscale (psychological health), IG participants experienced a significant improvement in AAQoL. Similar improvements in QoL have been observed in other studies on psychoeducational programmes for children [64] and adults [50, 65]. The following two factors could explain the absence of significant changes in other subscales in this study. First, the intervention duration, comprising two sessions over two weeks, may have been too brief to produce noticeable improvements in overall QoL. Second, the AAQoL scale in this study might not have been sensitive enough to detect minor changes in all subscales. Therefore, future RCT studies should explore how different quality-of-life scales can effectively measure improvements in brief educational group interventions.

Data collection during the post-intervention phase presented challenges. The scales were mailed to the outpatients, but some respondents who returned them in person to the clinic mentioned that they could have easily forgotten to mail the envelopes back. Although we followed up with nonresponders via social messaging services, future studies would benefit from improved data collection procedures. Furthermore, in future studies, having a research nurse on staff or implementing electronic surveys could help prevent missing data when collecting PROMs.

### Strengths and limitations

The primary strength of this study is developing a clinically relevant, person-centred educational programme for adults with ADHD. A secondary strength concerns involving user representatives and peer educators in creating, delivering and analysing the educational intervention. This study is a critical contribution to user involvement in nursing and mental health research. Moreover, this study involved a caregiver representative in the study design. The study measured the satisfaction of caregivers and outpatients, indicating excellent benefits for participants. Another strength is the ability to run a pilot feasibility RCT. Despite the small sample size, this pilot RCT provided promising preliminary findings for nurses, social workers and researchers, paving the way for full-scale RCT studies. Another strength is that most self-reported scales for measuring self-efficacy, QoL and satisfaction were validated; however, the three-item scale measuring patient satisfaction with the provided information was not validated.

Regarding the limitations, although randomisation equalises the treatment, it does not ensure a precise population estimate of the treatment effect. Moreover, randomisation does not remove the problem of unobserved covariates explaining the results (e.g. the severity and duration of the disorder, a satisfactory or poor response to ADHD medication, and the degree and quality of family members’ contact with the patient). Such covariates are likely to be evenly distributed in the groups due to randomisation, but further research should consider this selection bias that can influence the results. Further, the sample size was small, which can contribute to measurement bias and reduce generalisability.

This study provides valuable insight from the caregivers’ perspective; however, the limitations must be explored in greater depth, particularly those related to caregivers. The single-centre recruitment is a significant limitation that could affect the generalisability and validity of the findings. The limited participation of caregivers in only one session may have influenced their satisfaction and willingness to return. This finding highlights the need for future studies to ensure more comprehensive caregiver involvement, which could provide a clearer understanding of their perspectives and needs.

Due to the lack of a blind randomisation process, potential biases could have been introduced. The participants in this pilot RCT study were aware of their group assignment, which might have influenced the dropout rates. Participant-level masking should be implemented to minimise this bias. Nevertheless, participant masking was not implemented, given the face-to-face nature of the educational programme. The ITT analysis was conducted with masked group assignments to minimise bias. Furthermore, the responsible author was blinded to group allocation in these ITT analyses. Applying masking to the PP analyses was not possible. Comparing an individual education programme to a group-based one could also influence outcomes and attribution bias. The CG outpatients may have dropped out if they perceived the individual education was not what they wanted, possibly increasing the dropout rate. In that case, dropout rates could have been skewed, influencing the overall findings. Nevertheless, the reported dropout rates in this study were low, and dropouts were similar in both groups despite these concerns. Thus, incorporating blinding in future studies would address these limitations. Future studies should compare psychoeducation programmes to explore their most beneficial elements for those newly diagnosed with ADHD, including variations in the number and content of sessions.

The generalisability of these findings may be restricted because all participants and their caregivers were recruited from one CMHC. Recruiting participants from multiple centres in future RCT studies would facilitate a more representative sample. The enthusiasm of clinicians and user representatives facilitating the teaching could also be a source of bias in this pilot study specific to this centre. High attendance rates may have been positively influenced by this engagement, which may not be replicable in other settings. Hence, performance bias may have occurred if the attitudes and behaviours of the study personnel affected participant responses or engagement levels. The observed high engagement, rather than the intervention itself, may reflect the enthusiasm of those delivering the programme; thus, its feasibility and acceptability could be overestimated. Future studies should ensure training consistency for facilitators and standardise the engagement level across sites. Future multicentre studies may create a more balanced and generalisable intervention evaluation to introduce diversity into the intervention settings.

Further, the findings focus only on baseline and post-data. A follow-up assessment could have provided further information on the potential efficacy of the programme in terms of long-term improvements. Therefore, future RCT studies should involve multiple sites, larger sample sizes and more extended follow-up periods to understand the programme effects fully.

## Conclusion

This study presents an innovative educational programme for adults newly diagnosed with ADHD. The preliminary findings suggest that this educational programme is feasible and well-received by outpatients and their caregivers. However, future RCT studies involving larger sample sizes and more extended evaluation periods are necessary to capture the effects of this programme fully regarding self-efficacy, symptom severity and QoL.

## Electronic supplementary material

Below is the link to the electronic supplementary material.

Supplementary Material 1

## Data Availability

The dataset used in this pilot RCT study is not publicly available due to ethical considerations. Access to the data is restricted to protect the privacy and confidentiality of the participants involved.

## Abbreviations

AAQoL: Adult ADHD Quality of Life Scale
ADHD: Attention deficit hyperactivity disorder
ASRS: Adult ADHD Self-Reported Scale
CG: Control group
CI: Confidence interval
CMHC: Community mental health centre
CONSORT: Consolidated Standards of Reporting Trials
CSQ-4: Four-item Client Satisfaction Questionnaire
ITT: Intention-to-treat (analysis)
GSE-6: General Self-Efficacy 6, abridged version for ADHD
IG: Intervention group
M: Mean
PP: Per protocol
PROMs: Patient-reported outcome measures
QoL: Quality of life
RCT: Randomised controlled trial
SCL-9: Hopkin’s Symptom-Checklist 9-item Scale
SD: Standard deviation
TAU: Treatment as usual
TIDieR: Template for Intervention Description and Replication
